# Editorial: Small molecule inhibitors targeting mammalian selenoprotein thioredoxin reductases (TXNRDs): Interactions, mechanisms, and applications

**DOI:** 10.3389/fmolb.2023.1141772

**Published:** 2023-02-02

**Authors:** Jianqiang Xu, Alexios Vlamis-Gardikas, Jianguo Fang

**Affiliations:** ^1^ School of Life and Pharmaceutical Sciences (LPS) and Panjin Institute of Industrial Technology (PIIT), Dalian University of Technology, Panjin, China; ^2^ Department of Chemistry, University of Patras, Rion, Greece; ^3^ State Key Laboratory of Applied Organic Chemistry & College of Chemistry and Chemical Engineering, Lanzhou University, Lanzhou, China

**Keywords:** thioredoxin reductase, small molecule, selenoprotein, inhibitor, redox regulation, reactive oxygen species, cancer therapy

Selenoproteins have the selenium (Se)-containing amino acid selenocysteine (Sec) at their redox active motifs (or key activity sites). They are involved in diverse functions in human development, physiology, health, and diseases (Arner and Holmgren, 2006). Usually, the Sec is an essential residue for selenoproteins’ functions. Thioredoxin reductases (TXNRDs) are selenoproteins that regulate the redox environment in cells. The pivotal antioxidant effect is achieved by supplying electrons from NADPH to TXNRD and then to thioredoxin (TXN) in a system widely known as the TXN system (Zhang et al., 2017; Gencheva and Arnér, 2021; Zhang et al., 2022). As a housekeeping property, TXNRDs keep TXN and thioredoxin-related protein of 14 kDa (TRP-14) reduced, that the latter may further interact with multiple downstream proteins to regulate their functions (Dagnell et al., 2013; Pader et al., 2014; Espinosa and Arnér, 2019). TXNRD is usually overexpressed in tumor tissues i) to maintain a constant supply of electrons *via* TXN to ribonucleotide reductase so that deoxyribonucleotides will be available for the increased demands of DNA synthesis of malignant cells and ii) to offset any resulting redox imbalances in the cytosol. Inhibition of TXNRD is thus an appealing strategy in cancer chemotherapy. The inhibition of TXNRD may further cause oxidative stress to tumor cells, resulting into cell death *via* different pathways. The increasing use of small molecules as inhibitors of cytosolic TXNRD1 and mitochondrial TXNRD2 have been disclosed in the past decades. Targeting TXNRDs may reverse the growth of numerous tumors, making TXNRDs attractive targets for cancer chemotherapy (Cai et al., 2012; Zhang et al., 2021a).

At present, most TXNRD inhibitors are electrophiles that work *via* interaction with the highly reactive Sec residues of the enzymes (Zhang et al., 2018; Xu and Fang, 2021). This mode of inhibition may lead to cross reactivity with the widely available thiol groups, leading to unspecific inhibition of TXNRD in bench research and unwanted side effects in clinical trials. Thus, novel strategies that may employ structurally diverse inhibitors with novel targeting mechanism are highly desirable (Li et al., 2018). The combination of TXNRD inhibitors with other molecules, might lower side effects and/or enhance efficacy of treatment. Although the importance of TXNRD inhibitors is being increasingly recognized, only a few pharmaceutical companies are actively involved in the further development of such inhibitors. A close collaboration of academia and industry would facilitate the translation of bench results to bedside trials, and would expedite the application of TXNRD inhibitors in treating cancer or other diseases.

The Research Topic in the Frontiers in Molecular Biosciences (FMB) of Frontiers Publishers aimed at a better understanding of the mechanisms and actions of small molecules inhibiting mammalian selenoprotein TXNRDs: Interactions, mechanisms, and applications. In this Research Topic, small molecules included natural products, their interacting mechanisms for the inhibition of TXNRD, their downstream signaling pathways, their effect to human diseases and the results of their use in clinical trials.

Liver fibrosis is a precedent in the progression of liver injury into cirrhosis or even liver cancer. The role of the TXNRD/TXN system in liver fibrosis is unknown. Wenxuan Jiao et al., reported therapeutic effects concerning the organic selenium compound Butaselen (BS) as an inhibitor of TXNRD on liver fibrosis by downregulation of the transforming growth factor-β1 (TGF-β1)/Smads Pathway. In the study, liver fibrosis models were established using male BALB/c mice through intraperitoneal injection of CCl_4_. The authors revealed that BS, an inhibitor of the TXN system, exerted significant therapeutic effect on CCl_4_-induced liver fibrosis in mice. Hepatic stellate cells (HSCs) were used to examine the action mechanisms of BS against the progression of liver fibrosis. BS not only inhibited the activation of HSCs but also induced HSC apoptosis by inhibiting the TXN system. Thereby, BS attenuated hepatic fibrosis through inhibition of the production of α-smooth muscle actin (α-SMA) and collagens by HSCs by downregulating the TGF-β1 expression and blocking the TGF-β1/Smads pathway. This study shows the great potential of inhibitors of TXNRDs as drugs for the clinical treatment of liver fibrosis.

Natural products frequently have unique physiological activities and new action mechanisms due to their structural diversity and novelty. They are considered an important source for the development of innovative drugs and lead compounds. Junmin Zhang et al., showed that inhibition of TXNRD by Santamarine conferred anticancer effects in HeLa cells. In this study, a knockdown for TXNRD and cell lines overexpressing TXNRD were employed. The activity of TXNRD regulated the physiological effect of Santamarine in cells. In brief, the natural product Santamarine inhibited TXNRD and weakened the antioxidative function of the enzyme in cells, resulting in a high-level accumulation of ROS that finally induced an oxidative stress-mediated apoptosis to HeLa cells. The authors unveiled TXNRD is a novel target of Santamarine, proposing thus a previously unrecognized mechanism to explain its anticarcinogenic properties. Their findings provide a basis for the further development of Santamarine as a potential cancer therapeutic agent.

Glioblastoma multiforme (GBM) is the most aggressive and common form of glioma. In these tumors, TXN and TXNRD are overexpressed to cope with high levels of ROS and resist chemotherapy and radiotherapy. Thus, tackling the activity of these enzymes is a potential strategy to reduce cell viability/proliferation and most importantly achieve tumor cell death. Vanessa Pires et al., unveiled that mercury (Hg) compounds were among the most effective inhibitors of TXNRD and TXN due to their high affinity for binding thiols and selenols. Organomercurials such as Thimerosal (TmHg) could effectively cross the blood-brain barrier (BBB), to reach effective concentrations for the treatment of GBM. TmHg and its metabolite ethylmercury (EtHg) were evaluated over the mouse glioma cell line (GL261): both TmHg and EtHg inhibited the TXN system, triggered the cellular oxidative stress, and induced apoptotic cell death at low concentrations. Their study indicated that EtHg and TmHg have the potential of a therapeutic approach against GBM.

Redox regulators such as TXN and TXNRD have proved of utmost significance for the regulation of the redox milieu in normal cell survival, proliferation, invasion, metastasis of cancers and chemotherapy. In the review of Mirna Jovanović et al., the role of the TXN detoxification system in cancer progression and resistance is presented. The authors discussed some studies where the highly active TXN system contributed to the poor response of drug treatment, thus making it an attractive target for the development of drugs-inhibitors to be used for chemotherapy. Excessive oxidative stress is a characteristic of highly proliferative, metabolically hyperactive cancer cells, which are forced to mobilize antioxidant enzymes to suppress the increase in free radicals and thus prevent irreversible damage and cell death. Components of the TXN system are involved in high-rate proliferation and activation of pro-survival mechanisms in cancer cells, particularly those facing increased oxidative stress. The review highlighted the importance of the TXN system in tumor progression, as well as in the detoxification and protection of cancer cells from oxidative stress and drug-induced cytotoxicity. The authors further emphasized the importance of developing novel multitarget therapies encompassing the inhibition of the TXN system to overcome limitations in the current treatment of cancers.

The above four contributions from diverse range of fields, have already been published by the Frontiers in Molecular Biosciences (FMB). FMB’s success is due to an effective collaboration of authors, reviewers, and a dedicated editorial team under the leadership of Editor-in-Chief. We three guest editors are confident that these articles will be of interest to wide readers of FMB and will provoke new discussions in developing novel small molecules and novel targeting anti-tumor strategies.

Last but not the least, we would like to express our great gratitude to all authors of this Research Topic for their invaluable scientific contributions. Simultaneously, we thank all comments and opinions from the reviewers that have improved the article quality and scientific perspective. We hold our faith in bioscience and look forward to serving the Frontiers journals and research contributions in the future. Finally, we would be most happy if people with further questions or suggestions, contacted the executive editor of FMB. Please feel free to do so, it will be most helpful in further improving the quality of this journal.
